# Can Music Reduce Stress and Anxiety in the Operating Room Team? Insights from a Cross-Sectional Study in Northern Italy Healthcare Services

**DOI:** 10.3390/nursrep14020082

**Published:** 2024-04-27

**Authors:** Ivan Rubbi, Anna Roveri, Gianandrea Pasquinelli, Claudia Cadas, Maicol Carvello, Roberto Lupo, Elsa Vitale, Petia Di Lorenzo, Nicola Sangiorgi, Luana Conte, Valeria Cremonini

**Affiliations:** 1 School of Nursing, University of Bologna, 48018 Faenza, Italy; ivan.rubbi2@unibo.it (I.R.); anna.roveri@studio.unibo.it (A.R.); claudia.cadas@auslromagna.it (C.C.); valeria.cremonini@auslromagna.it (V.C.); 2 Department of Medical and Surgical Sciences, University of Bologna, 40126 Bologna, Italy; gianandr.pasquinelli@unibo.it; 3 “Community Hospital”, Local Health Authority (ASL) of Romagna, 48121 Ravenna, Italy; maicol.carvello2@unibo.it; 4 “San Giuseppe da Copertino” Hospital, Local Health Authority (ASL) of Lecce, 73043 Copertino, Italy; roberto.lupo@uniba.it; 5 Local Health Authority (ASL) of Bari, 70126 Bari, Italy; vitaleelsa@libero.it; 6 Multidisciplinar DH, Local Health Authority (AUSL) of Romagna, 48121 Ravenna, Italy; petia.dilorenzo@auslromagna.it (P.D.L.); nicola.sangiorgi@auslromagna.it (N.S.); 7 Laboratory of Biomedical Physics and Environment, Department of Mathematics and Physics “E. De Giorgi”, University of Salento, 73100 Lecce, Italy; 8 Advanced Data Analysis in Medicine (ADAM), Laboratory of Interdisciplinary Research Applied to Medicine (DReAM), Local Health Authority (ASL) Lecce and University of Salento, 73100 Lecce, Italy

**Keywords:** music, stress, anxiety, operating room

## Abstract

Background. Music evokes positive emotions and reduces stress and anxiety. Operating room (OR) staff face various challenges which can lead to high levels of stress. The aim of the study is to assess whether listening to music during intraoperative phases improves the work environment by reducing anxiety and stress in the entire surgical team. Methods. A prospective observational study was conducted from February to September 2023, involving medical personnel, nursing staff, and nursing students. They were divided into two groups: Group 1 with music during surgical procedures, and Group 2 without music. Participants were administered two validated instruments: the Zung Anxiety Self-Assessment Scale (SAS) to measure anxiety, and the Positive and Negative Affect Schedule to assess emotions generating stress. Additional items were included for demographics, job satisfaction, and the organization method. Results. Music did not impact anxiety, but increased positive emotions while reducing negative ones. Music had an ancillary effect, highlighting the need for significant organizational interventions aimed at increasing operator satisfaction, including offering voluntary instead of mandatory assignments to nursing staff. Conclusions. Music appears to reduce stress in the intraoperative team when supported by a positive work environment in which assigned operators have chosen to work in the OR.

## 1. Introduction

Music has the ability to evoke and regulate emotions, provide pleasure and comfort, and alleviate stress [1]. Several studies demonstrate that, in addition to reducing stress, music also reduces anxiety, improves sleep quality, decreases fatigue, enhances well-being, and alleviates pain [2,3,4,5]. Listening to music increases coping abilities and promotes relaxation after stressful events [6,7].

The use of music in workplaces has proven effective in reducing tension and improving mental state, performance, attention, and concentration. However, attention to volume and melody rhythm is crucial [8,9]. Through music, there is a reduction in fatigue during work activities [10].

Surgical intervention is a stressful exercise that requires the expert execution of techniques and non-technical skills such as communication, teamwork, and quick decision-making under pressure [11]. In addition to these factors, there are often long working hours for operators, the need to treat patients with severe clinical conditions, and significant consequences in the event of an error. These situations can lead to high levels of stress [12]. Stress and the management of its effects on performance are common challenges for all operating room professionals [11]. Elevated perceived stress levels during surgical procedures not only negatively affect healthcare professionals but can also compromise patient safety and the quality of care [13]. Several studies indicate that patient safety failures result from human failures related to communication, teamwork, and the psychological health of professionals [14,15,16]. Professional well-being, anxiety, and stress management are among the determining factors that influence patient care [17,18].

Music, with its benefits, is significantly used in operating rooms worldwide to positively modify the environment where surgeons and the entire healthcare staff operate [11,19,20]. However, the literature is not yet unanimous on the benefits that music can have in these contexts. Some studies suggest that music has a distracting effect, especially in critical moments, associated with a reduction in auditory perception and speech [21,22], and an increase in the frequency of repeated requests [23] inhibits the ability to perform tasks safely and poses a threat to the health and safety of both patients and staff [24]. In addition, some believe that music may “mask” alarms [25]. Conversely, other studies argue that music is generally a favorable part of the operating room environment [10,26,27], as it seems to improve calmness [19], stress autonomic reactivity [25,28], mood, and the performance of the surgeon and the entire assistive team [11,26]. Recent systematic reviews have highlighted that background music can improve the accuracy and speed of surgical interventions [9], reducing mental workload [29]. For these reasons, a significant portion of nursing and medical staff believes that they appreciate their work more and achieve better results when music is played in the operating room [26]. Other research suggests that the aforementioned ‘benefits’ of music played in the operating room are more conjecture than fact; the reality is that it is a widely accepted cultural practice [30]. The word ‘cultural’ is also used to indicate that the type of music played often reflects the demographics of the surgeon [31].

The use of music as a tool of interest in managing stress and associated health issues must be measured [11]. Multiple validated questionnaires in the literature allow for investigating stress and anxiety levels in workplaces. The “Zung Anxiety Self-Assessment Scale” is a clinical tool used to analyze subjective anxiety [32], while the “Positive and Negative Affect Schedule” evaluates the positive and negative emotions of participants that typically generate stress [33].

Objectives: The purpose of the study is to describe whether there are differences among the staff who use or do not use music in the operating room, measuring anxiety and performance stress as parameters.

## 2. Methods

### Study Design: Prospective Cross-Sectional Study

Participants: Non-probabilistic sample. Availability to participate in the study on a voluntary basis was requested from operating room staff, including anesthesiologists, surgeons of various specialties, scrub nurses, circulating nurses, and nursing students on placement. Operating rooms in three hospitals affiliated with a healthcare company in Northern Italy were recruited. The three hospitals enrolled in the study included a major facility with 12 operating rooms where primarily high-complexity major surgeries were scheduled, and two other facilities of lower complexity with fewer operating rooms: one with 9 rooms and the other with 4 rooms. Multispecialty surgeries were guaranteed in all hospitals. The sample was divided into two groups: Group 1 listened to music during surgical procedures, and Group 2 did not use music during operating room activities.

Setting: The groups were divided based on the organizational model adopted by the various operating rooms in the three hospitals. Not all operating rooms use music; therefore, two groups were created.

For the staff in Group 1, the music they listened to was 80% Italian light music and 20% international light music. Music was played from 9:30 am until the end of the surgical procedure. Music was selected and controlled by the room nurse with the audio volume set so as not to hinder staff communications (<60 dB) [8,9,27], using online streaming music channels [31]. Music was not activated during the anesthesiology phase when the patient was being put to sleep [24] but only afterward during the surgical phase for the entire procedure. Based on the type of performance (e.g., suturing), the surgeon would express their musical preference and/or switch from light music to something more rhythmic [31]. Data were extracted from a database created through the survey, following the administration of the questionnaire. The staff completed the questionnaire on a platform through an access link provided by the researcher physically present in the operating rooms, who monitored the correct procedure and the timing of filling out the instrument. The questionnaire was always completed at the end of the surgical procedures. Anonymity was safeguarded for the sample, and responses could not be associated with individual professionals in any way. Emergency surgical interventions were excluded from the study.

The study focused on professionals conducting or assisting operations on patients from February to September 2023.

Instruments: Data were collected through the Zung Anxiety Self-Assessment Scale [32,34] and the Positive and Negative Affect Schedule (PANAS-SF) [33].

The first questionnaire measures anxiety levels for those exhibiting anxiety-related symptoms. Each response uses a Likert scale ranging from 1 to 4 points, where 1 corresponds to “never” and 4 corresponds to “most of the time or all the time”. There are 20 questions, and the total score is obtained by summing the scores for each response. Each possible total score corresponds to a numerical score defined as the “Anxiety Index”, which varies based on the total score. The Anxiety Index is determined based on the Likert total score on a range from 20 to 100: Normal = <45; Minimal = 45–59; Severe = 60–74; Extreme = >75 [32].

The second questionnaire is one of the most widely used tools for assessing positive and negative affective states. The questionnaire consists of 20 adjectives, divided into two sections, with 10 composing the positive affect scale and 10 the negative affect scale. The positive affect section reflects the degree to which a person feels enthusiastic, active, and determined. The negative affect section refers to general unpleasant states such as anger, guilt, and fear. For each proposed item, respondents were asked to evaluate how closely that adjective reflected their mood at the time of the intervention, reporting the data on a 5-point Likert scale, where 1 indicates “very little or not at all” and 5 indicates “extremely”. To interpret the results, scores were summed for each positive and negative affect term. Lower scores indicate lower levels of affect, while higher scores represent higher levels of affect. Watson et al. indicate an average score of 33.3 ± 7.2, while for the negative section, we have an average score of 17.4 ± 6.2 [33].

In the demographic data section, questions were included on gender, age, profession, years of service in the operating room, and satisfaction with the work environment [35]. For nursing staff, questions included post-basic training and whether assignment to the operating room was voluntary or mandatory.

A 15 min time allocation was provided for questionnaire completion.

Bias: To avoid response bias during questionnaire completion, the researcher physically visited hospital facilities and entered the designated relaxation areas for operating room staff. During the informed consent phase, basic computer literacy training was provided to participants on the correct use of the platform and the questionnaire characteristics [36]. This approach facilitated consent and helped reduce missing responses [37]. Using a tablet, participants were given ample time to reflect in order to obtain more accurate responses [37]. The researcher ensured that staff were not subjected to performance-related variables (e.g., completing healthcare documentation or answering phone consultations) [38].

Statistical analysis: The collected data were organized using an electronic database and analyzed with the statistical software Jamovi 2.3.18. Descriptive statistical (mean, median, standard deviation, frequencies, and percentages), T-tests, and ANOVA analyses were conducted to identify significant differences using a 95% confidence interval. The internal consistency of both instruments was assessed using Cronbach’s Alpha, and the sample adequacy was measured with the Kaiser–Meyer–Olkin (KMO) statistic. The multiple comparison was conducted using Tukey’s HSD.

Ethical considerations: The study received approval from the Ethics Committee (Protocol 0026393 dated 2 February 2023) and the Health Directorate of the Company. The study was conducted in accordance with the Declaration of Helsinki.

## 3. Results

Overall, 122 professionals participated in the study, including 29.5% (*n* = 36) surgeons with an average age of 42.6 ± 8.33, 11.5% (*n* = 14) anesthetists with an average age of 39.2 ± 4.85, 22.1% (*n* = 27) instrument nurses with an average age of 43.1 ± 11.4, 11.5% (*n* = 14) anesthesia nurses with an average age of 46.1 ± 9.45, 13.1% (*n* = 16) ward nurses with an average age of 44.4 ± 10.5, and 12.3% (*n* = 15) nursing students with an average age of 23.1 ± 2.92. Regarding gender, 56.6% (*n* = 69) were female, and 43.4% (*n* = 53) were male.

Regarding years of experience in the operating room, the mean values were approximately 16.0 ± 11.1 for surgeons, 8.7 ± 5.28 for anesthetists, 15.3 ± 12.2 for instrument nurses, 14.5 ± 13.1 for anesthesia nurses, and 18.0 ± 11.2 for ward nurses. The students recorded an average experience of 1.5 months.

For nursing professionals, the study inquired about post-basic training related to the operating room. A total of 7.0% (*n* = 4) reported not having post-graduate training, 36.8% (*n* = 21) indicated having a Master’s degree, and 56.2% (*n* = 32) reported having company-specific training related to their role. Among nurses, 82.5% (*n* = 47) chose to work in the operating room voluntarily, while 17.5% (*n* = 10) were assigned to the role.

Regarding the division of the sample into groups, 66.4% (*n* = 81) belonged to Group 1 (exposed to music) and 33.6% (*n* = 41) belonged to Group 2 (not exposed to music). The selection of groups was based on the organizational models that either included or did not include the use of music in the operating room.

Internal consistency was acceptable for SAS (α = 0.682) and good for PANAS-SF (α = 0.834). Sample adequacy was good for both instruments [SAS (KMO = 0.725); PANAS-SF (KMO = 0.825)].

In Table 1, although not statistically significant, PANAS-SF (Positive Score) indicates a positive effect on subjective well-being in the operating room professionals with music (34.2 ± 6.14) compared to those without music (32.9 ± 8.38), (Δ = +1.3). Similarly, for anxiety, which is normal for both settings and lacks significance, Group 1 recorded a lower value with 36 vs. 39. Regarding the negative effects of PANAS-SF (Negative score), the value is below the average score for both settings; however, Group 1 shows a slightly higher average score (15.4 ± 5.58) compared to Group 2 (14.3 ± 4.94), (Δ = +1.1) (*p* = 0.292).

In Table 2, the SAS showed normal anxiety levels for all professionals in both settings, with no significant differences. However, in the first group, values below 40 on the index were recorded in medical staff, instrument nurses, ward nurses, and students. The highest value was recorded in anesthesia nurses, with an index of 41. In Group 2, with the index value below 40, we find doctors, anesthesia nurses, and ward nurses. Instrument nurses and students recorded scores above 40.

Regarding the Positive Affect Score, no significant differences were recorded within either group in terms of feelings of enthusiasm, determination, or activism. However, in Group 1, it is the surgeons who record the highest score (36.1 ± 6.08), followed by the students (35.2 ± 7.24), anesthesiologists (33.9 ± 5.85), instrument nurses (32.8 ± 6.18), operating room nurses (32.4 ± 5.45), and finally, anesthesia nurses (31.4 ± 5.52).

In Group 2, the number of doctors (surgeons and anesthesiologists) is lower compared to Group 1. Specifically, in the first group, there are 29 surgeons compared to 7 in the second group, and 12 anesthesiologists compared to 2 in Group 2. As for the nurses, the instrument nurses in Group 1 are 13 versus 14 in Group 2, while the operating room nurses are 8 in both groups. The anesthesia nurses are 10 in the first group versus 4 in the second group. For this reason, when comparing the two groups by individual operators, the nurses in Group 1 record higher scores compared to their colleagues in Group 2: instrument nurses Δ = +1.2; operating room nurses Δ = +3.6; anesthesia nurses Δ = +3.1.

PANAS-SF’s Negative Affect Score in Group 1 highlighted a value of 17.6 ± 5.32 among anesthesia nurses with a Δ (+3.3) compared to colleagues with the same role in Group 2 (Table 2).

The assignment mode in the operating room revealed a statistically significant difference in the negative effects measured using PANAS. The nursing staff assigned involuntarily, compared to those with voluntary assignment, recorded a Negative Affect Score with a mean of 19.0 ± 6.50 and a median of 21.0, *p* = 0.027 (Table 3).

The level of satisfaction for the work performed shows significant differences only in the Positive Affect Score (*p* = 0.003), with a score which increases proportionally as the Likert score increases (Table 4). The post hoc analysis using Tukey’s HSD revealed a significant difference at the Likert score of 3 compared to scores of 4 [Mean difference = −4.190 (*p* = 0.032)] and 5 [Mean difference = −5.583 (*p* = 0.004)].

The results from Table 4 are confirmed by the Pearson correlation index reported in Table 5. The Negative Affect Score (−0.243) shows a negative correlation with the satisfaction that professionals perceive about their work environment (*p* < 0.01), while the Positive Affect Score shows a significant positive correlation of 0.313, (*p* < 0.001). The Anxiety Index Scale is positively correlated with the Negative Affect Score (0.501; *p* < 0.001).

Other independent variables, such as the post-basic training received by nurses, did not show significant differences in the Anxiety Index (F = 0.64; *p* = 0.54), Positive Affect Score (F = 3.203; *p* = 0.07), or Negative Affect Score (F = 2.402; *p* = 0.14).

The only indicator that yielded a positive result in the Anxiety Index (Index = 50; Raw Score = 40) pertains to married operators. In comparison to others with a normal index, they exhibit a statistically significant difference (F = 6.356; *p* = 0.002).

## 4. Discussion

The use of music in the operating room appears to promote positive emotions and reduce stress among healthcare professionals [26]. Overall, the study did not reveal substantial differences in anxiety and stress between the group that uses music in the operating room and the one that does not. The anticipated benefits of music appear to be more conjectural than factual [30]. However, the study found that the choice of music by the staff, especially the surgeons, based on the performance activities carried out, resulted in a significant Positive Affect Score compared to Group 2. This probably aligns with what Butler et al. [31] defined as a cultural practice, leading to a Positive Affect correlated to the musical tastes of the staff. Indeed, within Group 1, despite the absence of statistical significance, the surgeons expressed higher levels of Positive Affect, exceeding 35.00. However, the significant difference compared to Group 2 is attributed to the overall score of the nurses who selected tracks based on their own personal musical preferences in the absence of specific directions from the surgeons. Regarding anxiety, the data from the study do not indicate states of anxiety in the healthcare personnel in either group, and for this reason, it was not possible to assess the positive effects of music as documented in the literature on patients [39]. Although lower Likert scores on the SAS were observed in Group 1 compared to Group 2, these differences were not statistically significant according to the *T*-test.

It is very likely that the substantially normal indicators of the SAS and the Negative Affect could be partly attributed to a very low percentage of dissatisfaction with one’s work in the operating room (<6%). This finding seems to be in agreement with Movahedi et al. [35], who demonstrated that the degree of professional satisfaction is considered an indicator of good organizational policy. Good organizational policy and satisfaction in the workplace appear to reduce anxiety and stress [40]. The results of this study seem to be consistent with the literature, which shows that satisfaction is directly proportional to the Positive Affect Score and inversely proportional to the SAS and Negative Affect Score.

Regarding the mode of listening to music, setting the audio volume to <60 dB is supported by the literature. Recent studies have shown that the volume of music can be the main source of distraction in the operating room, rather than the mere presence of music [41].

In a study evaluating the performance of surgeons performing laparoscopic skills in various environments (quiet, noise at 80–85 dB, and music), no difference in their performance was observed [42]. However, other studies link noise to increased cortisol levels in patients, which subsequently leads to higher rates of postoperative infections [27]. In a study conducted by Hamad et al. [43], 60.5% of the operating room staff perceived music as noise, with volume ranging from 59.52 to 85.60 dB. For this reason, the fact that the anesthesiologists and anesthesia nurses involved in the study express the need to not have background music during the administration of anesthesia seems to be confirmed by the literature. Indeed, a systematic review has shown that environmental distractions due to excessive noise in the operating room during anesthesiological phases can decrease vigilance and potentially delay the recognition of non-routine events. However, during less active parts, music can help anesthesiologists and nursing staff remain alert and might reduce instances of irrelevant conversation, which is often cited as a distraction factor in the operating room [44]. Research thus seems to frame the use of music as a beneficial intervention associated with a positive work environment where staff are selected not only based on skills but also on the motivation to work in operating units. This combination not only enhances job satisfaction but also has promising implications for managing stress effectively in the operating room.

## 5. Limitations

This study is not without methodological limitations. The first limitation relates to the sample size; indeed, it would be useful to replicate the study in multiple healthcare companies nationwide. A second limitation could be attributed to the sample selection method. Voluntary participation is likely to introduce selection biases, as those who chose to participate might have different perspectives and experiences compared to those who chose not to participate.

## 6. Conclusions

This study demonstrates that music might have a modest positive effect on the subjective well-being of healthcare workers in the operating room, although this effect is not sufficient on its own. This research did not find significant differences in anxiety and stress levels between staff who use music and those who do not. The literature suggests that efforts should also focus on enhancing motivation, satisfaction, and the work climate of professionals to potentially amplify the benefits.

## Figures and Tables

**Table 1:** Comparison between groups using SAS and PANAS-SF.

	Group 1 (Exposed to Music) *N* = 81		Group 2 (Not Exposed to Music) *N* = 41			
	Point Likert	Index	Point Likert	Index		
	M ± SD (Median)		M ± SD (Median)		t	*p*
SAS (Anxiety Index)	30.0 ± 5.42 (29.0)	37.6 ± 6.74 (36.0)	30.6 ± 5.94	38.4 ± 7.46 (39.0)	0.637	0.525
PANAS-SF (Positive Affect Score)	34.2 ± 6.14 (35.0)	++	32.9 ± 8.38	-	−0.952	0.343
PANAS-SF (Negative Affect Score)	15.4 ± 5.58 (14.0)	-	14.3 ± 4.94	-	−1.058	0.292

++ (Positive Affect) = > 33.3; - (Positive Affect and Negative Affect) = < 33.3 and < 17.4; M ± SD = Mean and standard deviation.

**Table 2:** Effects of music on anxiety and stress in different profiles in the operating room.

		Group 1				Group 2			
	Sample	N	Anxiety Index M ± SD (Median)	F	*p*	N	Anxiety Index M ± SD (Median)	F	*p*
SAS	Surgeon	29	38.6 ± 5.75 (38.0)	0.554	0.734	7	32.9 ± 8.03 (30.0)	1.14	0.420
	Anesthetist	12	35.7 ± 6.11 (35.5)			2	37.5 ± 12.02 (37.5)		
	Instrument Nurse	13	38.3 ± 8.87 (36.0)			14	42.1 ± 6.98 (43.5)		
	Anesthesia Nurse	10	38.5 ± 7.26 (41.0)			4	35.5 ± 7.23 (34.5)		
	Operating Room Nurse	8	35.8 ± 7.44 (36.0)			8	38.4 ± 5.68 (39.0)		
	Student	9	36.6 ± 6.73 (35.0)			6	38.5 ± 6.92 (40.5)		
		**Group 1**				**Group 2**			
		N	M ± SD	F	*p*	N	Media	F	*p*
PANAS-SF (Positive Affect Score)	Surgeon	29	36.1 ± 6.08	1.271	0.306	7	38.0 ± 9.87	1.589	0.274
	Anesthetist	12	33.9 ± 5.85			2	37.0 ± 12.73		
	Instrument Nurse	13	32.8 ± 6.18			14	31.6 ± 6.92		
	Anesthesia Nurse	10	31.4 ± 5.52			4	28.3 ± 5.56		
	Operating Room Nurse	8	32.4 ± 5.45			8	28.8 ± 9.39		
	Student	9	35.2 ± 7.24			6	37.3 ± 5.47		
PANAS-SF (Negative Affect Score)	Surgeon	29	15.8 ± 6.96	2.335	0.069	7	11.1 ± 3.02	1.169	0.402
	Anesthetist	12	12.8 ± 2.25			2	12.5 ± 3.54		
	Instrument Nurse	13	16.7 ± 5.41			14	15.2 ± 6.28		
	Anesthesia Nurse	10	17.6 ± 5.32			4	14.3 ± 2.87		
	Ward Nurse	8	14.0 ± 4.47			8	14.9 ± 4.97		
	Student	9	14.3 ± 4.42			6	15.7 ± 4.37		
ASAS: Normal = < 45; Minimal = 45–49; Severe = 60–74; Extreme = > 75									
PANAS-SF Positive Affect Score: M ± SD (33.3 ± 7.2)									
PANAS-SF Negative Affect Score: M ± SD (17.4 ± 6.2)									

M ± SD = Mean and standard deviation.

**Table 3:** Values of SAS and PANAS based on nurses’ assignment modes.

	Voluntary Assignment		Office Allocation			
	Median	M ± SD	Median	M ± SD	t	*p*
SAS (Anxiety Index Scale)	44.5	42.1 ± 8.14	38.0	38.0 ± 7.16	1.606	0.114
PANAS-SF (Positive Affect Score)	32.0	31.7 ± 6.72	29.5	29.5 ± 5.62	−0.974	0.334
PANAS-SF (Negative Affect Score)	14.0	15.0 ± 4.73	21.0	19.0 ± 6.50	2.280	0.027 *

* *p* = <0.05; M ± SD = Mean and standard deviation.

**Table 4:** Values of SAS and PANAS based on job satisfaction of personnel.

	2	3	4	5		
	*n* = 6	*n* = 34	*n* = 46	*n* = 35	F	*p*
SAS (Anxiety Index Scale)					1.450	0.232
Median	38.5	38.5	36	36.0		
M ± SD	42.0 ± 7.72	38.6 ± 6.75	37.3 ± 7.06	36.5 ± 5.90		
PANAS-SF (Positive Affect Score)					4.881	0.003 **
Median	29.0	31.5	36.0	36.0		
M ± SD	30.7 ± 7.89	30.6 ± 7.07	34.8 ± 6.76	36.2 ± 5.90		
PANAS-SF (Negative Affect Score)					2.486	0.064
Median	13.0	15.0	13.0	11.0		
M ± SD	17.3 ± 8.36	16.3 ± 4.89	15.0 ± 6.02	13.2 ± 3.54		

** *p* < 0.01; M ± SD = Mean and standard deviation.

**Table 5:** Pearson correlation.

	“Express Your Level of Satisfaction Regarding Your Job Position”	Anxiety Index Scale
SAS (Anxiety Index Scale)	−0.242 **	—
PANAS-SF (Positive Affect Score)	0.314 ***	−0.089
PANAS-SF (Negative Affect Score)	−0.278 **	0.522 ***

** *p* < 0.01; *** *p* < 0.001.

## Data Availability

Data will be available upon request.

## Public Involvement Statement

Participants in the study were healthcare professionals from three hospitals affiliated with a healthcare company in Northern Italy. Consent was obtained from all participants.

## Guidelines and Standards Statement

This manuscript was drafted based on the Strengthening the Reporting of Observational Studies in Epidemiology (STROBE) guidelines for cross-sectional studies.
